# Supplementary material for: EVL regulates VEGF receptor‐2 internalization and signaling in developmental angiogenesis
Source: EMBO Rep. 2021 Jan 29;22(2):e48961. doi: 10.15252/embr.201948961 (PMC7857432; doi:10.15252/embr.201948961)
Supplement: Supplementary file 1 — Expanded View Figures PDF [file EMBR-22-e48961-s001.pdf]

Expanded View Figures

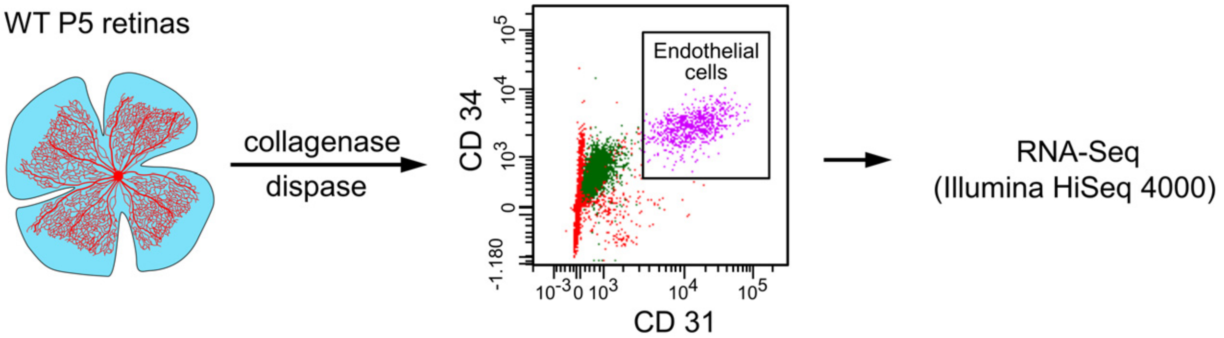

Figure EV1. Overview of retinal endothelial cell isolation for RNA sequencing.
P5 retinas from wild-type mice were digested with collagenase and dispase. CD31 and CD34 double-positive endothelial cells were isolated by FACS, pooled into two samples, and analyzed by RNA-Seq on an Illumina HiSeq 4000 sequencer generating 50 bp single-end reads (*ca.* 30–40 Mio reads/sample).

 © 2021 The Authors

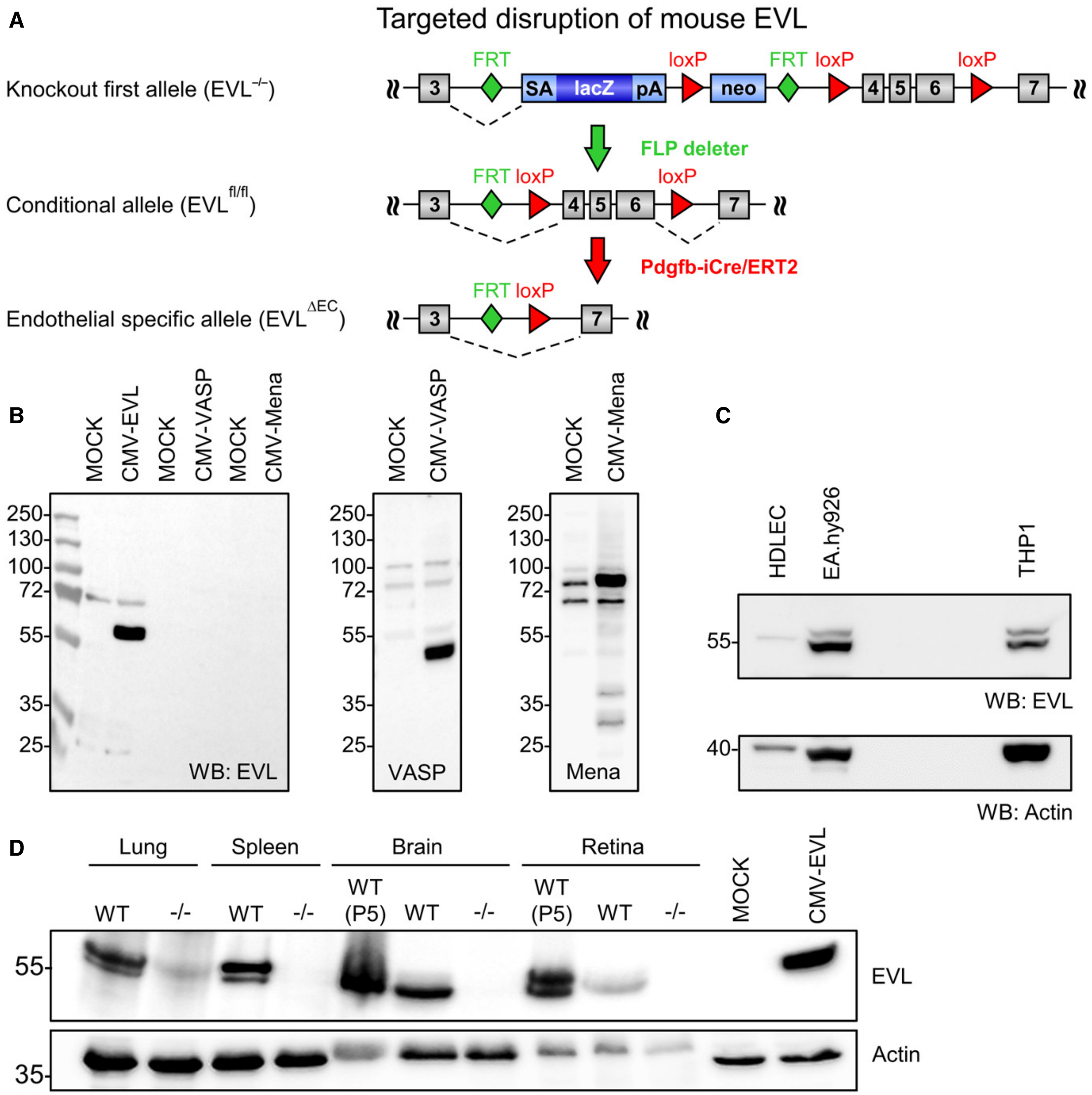
A
Targeted disruption of mouse EVL
Knockout first allele (EVL−/−)
FRT
loxP
FRT loxP
loxP
3
SA lacZ pA
neo
4 5 6
7
FLP deleter
Conditional allele (EVLfl/fl)
Pdgfb-iCre/ERT2
Endothelial specific allele (EVLΔEC)
B
MOCK
CMV-EVL
CMV-VASP
CMV-Mena
WB: EVL
VASP
Mena
C
HDLEC
EA.hy926
THP1
WB: EVL
WB: Actin
D
Lung
Spleen
Brain
Retina
WT
-/-
WT (P5)
MOCK
CMV-EVL
EVL
Actin

Figure EV2.

Figure EV2. Targeted disruption of the mouse EVL gene.

A Generation of global and tissue-specific EVL-deficient mice. In the knockout first alleles, a trapping element consisting of a splice acceptor (SA), the promoterless lacZ gene, a polyadenylation signal (pA), and a neomycin resistance (neo) is inserted in intron 3 of the EVL gene. Splicing (dashed line) from EVL exon 3 (gray box) to the splice acceptor of the trapping cassette induces the global disruption of the EVL gene ($EVL^{-/-}$). FLP-mediated recombination of the FRT sites (green rectangles) deletes the trapping elements and generates conditional alleles ($EVL^{fl/fl}$) with loxP sites flanking the critical EVL exons 4–6. Recombination of loxP sites (red triangles) by the tamoxifen-inducible, pdgfb-driven Cre (Pdgfb-iCre/ERT2) deletes EVL exons 4–6, creates a frameshift mutation, and thus generates endothelial-specific EVL-deficient mice ($EVL^{\Delta EC}$).

B Characterization of EVL-specific antibodies. HEK293 cells were transfected with EVL, VASP, or Mena (CMV-EVL, -VASP, -Mena) or MOCK (CMV-expression plasmid without insert) and analyzed by Western blotting with EVL-specific antibodies (left panel). Expression of VASP and Mena in the corresponding lysates was confirmed by Western blotting with anti-VASP (middle) or anti-Mena (right) antibodies, respectively.

C Primary human dermal lymphatic endothelial cells (HDLEC), human endothelium-derived cells (EA.hy926), and human monocytic cells (THP-1) were lysed and analyzed by Western blotting with EVL-specific antibodies (upper panel) or actin-specific antibodies (lower panel).

D Western blot analysis of EVL expression in lung, spleen, brain, and retina from adult wild-type (WT), P5 wild-type (WT P5), and adult $EVL^{-/-}$ (−/−) mice. EVL-specific antibodies detected the short EVL (60 kDa) and the long EVL-I (65 kDa) protein isoforms. MOCK and EVL-transfected HEK cells were used as positive or negative controls, respectively. Actin was used as loading control.

Figure EV3. Subcellular localization of EVL and postnatal angiogenesis in $VASP^{-/-}$ mice.

A EVL localizes to focal adhesions in endothelial cells. MLEC from wild-type mice were stained for phospho-paxilin (green) as a marker for focal adhesions, EVL (red) and actin (blue). White arrows indicate integrin-based focal adhesions at the tips of actin stress fibers. Representative images from three independent experiments are shown. Scale bar, 10 μm.

B, C Postnatal retinal angiogenesis in $VASP^{-/-}$ mice. (B) Isolectin B4-stained vasculature in whole mount retinas of wild-type (WT) and global $VASP^{-/-}$ mice on postnatal days 3 and 5 (P3, P5) assessed by confocal microscopy. Scale bars 200 μm. (C) Analysis of the radial vascular outgrowth relative to retinal radius and normalized to wild-type littermates. Error bars represent SEM; no significant difference was observed between the two genotypes at P3 ($P > 0.999$) or P5 ($P > 0.999$) (one-way ANOVA with Bonferroni's multi-comparison test).

 © 2021 The Authors

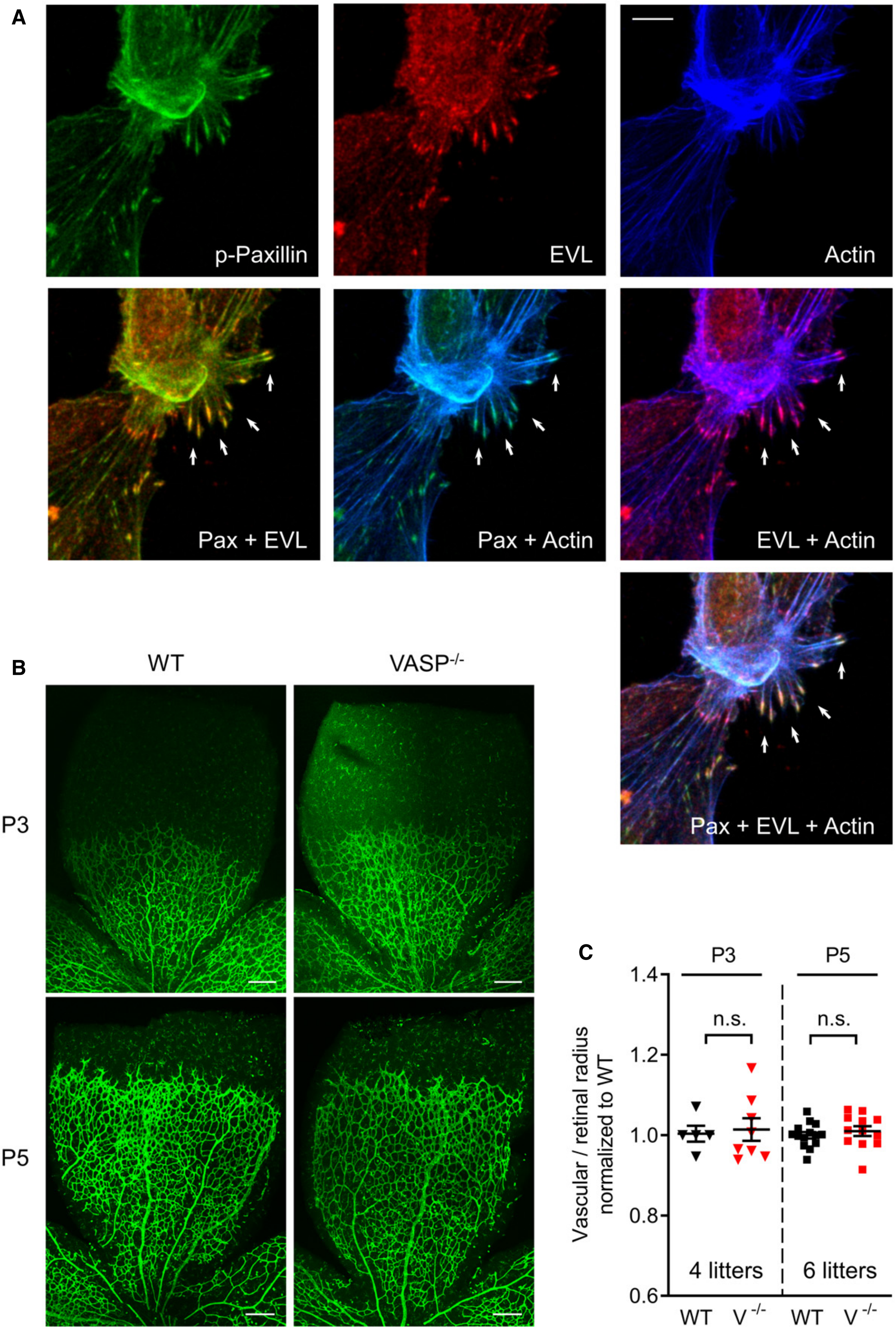
A
p-Paxillin
EVL
Actin
Pax + EVL
Pax + Actin
EVL + Actin
Pax + EVL + Actin
B
WT
VASP-/-
P3
P5
C
P3
P5
n.s.
n.s.
Vascular / retinal radius normalized to WT
1.4
1.2
1.0
0.8
0.6
4 litters
6 litters
WT
V -/-
WT
V -/-

Figure EV3.

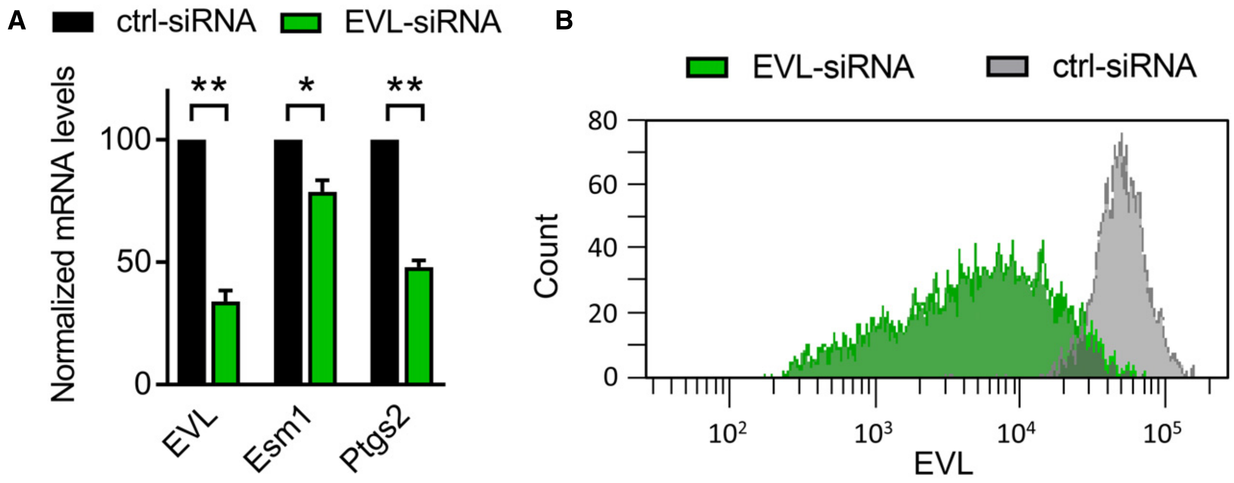

Figure EV4. mRNA analysis of EVL siRNA transfected HUVEC.

A Analysis of mRNA levels of EVL, Esm1, and Ptgs2 in HUVEC transfected with EVL-specific or control siRNA. $n = 3$ independent experiments, error bars represent SEM, one-sample t-test, $^*P < 0.05$; $^{**}P < 0.01$.

B FACS analysis of EVL protein expression in HUVEC transfected with EVL-specific or control siRNA. One representative image of three independent experiments is shown.

 © 2021 The Authors

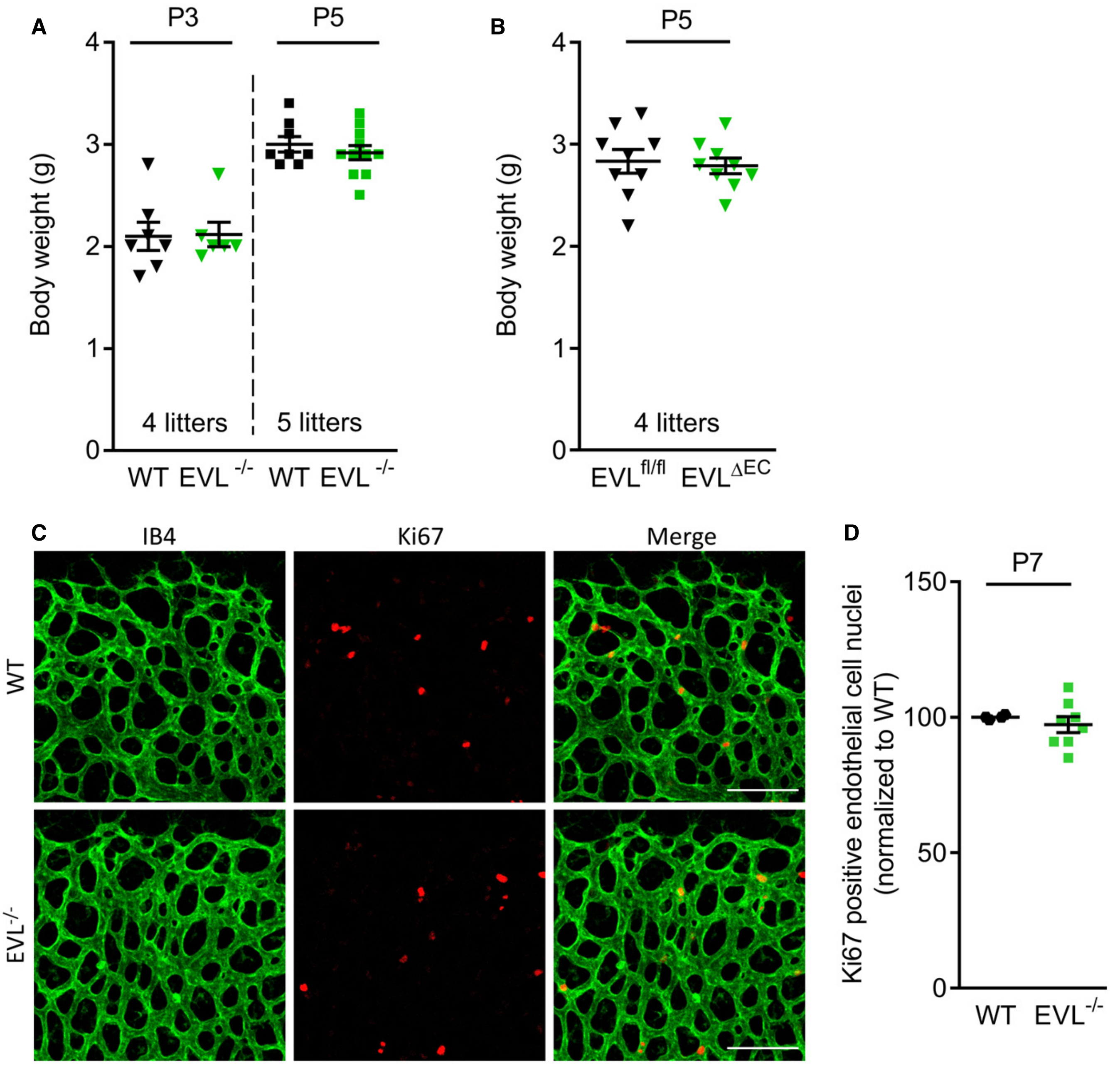

Figure EV5. Body weight of EVL-deficient mice and proliferation of EVL$^{-/-}$ retinal endothelial cells at P7.

A, B Body weight of wild-type and EVL-deficient mice. (A) Body weight of wild-type and global EVL-deficient mice at postnatal days 3 and 5. No significant difference in body weight was observed between the two genotypes at P3 ($P > 0.999$) or P5 ($P > 0.999$). One-way ANOVA with Bonferroni's multi comparison test; 4 and 5 different litters, respectively, error bars represent SEM. (B) Endothelial cell-specific EVL knockout mice (EVL$^{\Delta EC}$) and littermate controls (EVL^{fl}/^{fl}) at P5. No significant difference in body weight was observed between the two groups ($P = 0.753$). Unpaired Student's *t*-test. Error bars represent SEM.

C Ki67 expression in P7 retinas of wild-type (WT) and global EVL$^{-/-}$ mice. Retinas were fixed and stained with isolectin B4 (IB4, green) to visualize endothelial cells and antibodies directed against Ki67 (red) to visualize proliferation. Representative images from three independent experiments are shown. Scale bars, 100 µm.

D Analysis of proliferation of wild-type and EVL$^{-/-}$ retinal endothelial cells at P7 by Ki67 immunofluorescence microscopy. Data are normalized to the mean WT value. No significant difference in Ki67-positive endothelial cells was observed between the two groups ($P = 0.386$). Unpaired Student's *t*-test; two litters. Error bars represent SEM.

© 2021 The Authors
